# Left Atrial Strain: State of the Art and Clinical Implications

**DOI:** 10.3390/jpm14111093

**Published:** 2024-11-05

**Authors:** Niloofar Javadi, Nadera N. Bismee, Mohammed Tiseer Abbas, Isabel G. Scalia, Milagros Pereyra, Nima Baba Ali, Sogol Attaripour Esfahani, Kamal Awad, Juan M. Farina, Chadi Ayoub, Reza Arsanjani

**Affiliations:** Department of Cardiovascular Medicine, Mayo Clinic, Phoenix, AZ 85054, USA; javadi.niloofar@mayo.edu (N.J.); bismee.naderanaquib@mayo.edu (N.N.B.); abbas.mohammedtiseer@mayo.edu (M.T.A.); scalia.isabel@mayo.edu (I.G.S.); pereyra.milagros@mayo.edu (M.P.); babaali.nima@mayo.edu (N.B.A.); attaripouresfahani.sogol@mayo.edu (S.A.E.); awad.kamal@mayo.edu (K.A.); farina.juanmaria@mayo.edu (J.M.F.); ayoub.chadi@mayo.edu (C.A.)

**Keywords:** left atrial strain, speckle-tracking echocardiography, cardiovascular diseases

## Abstract

The assessment of left atrial strain (LAS) has emerged as an essential component in the evaluation of cardiac function, especially in pathologies such as heart failure and atrial fibrillation. This narrative review aims to outline the available methods for assessing LAS with a major emphasis on speckle-tracking echocardiography techniques. Other imaging modalities, including cardiac magnetic resonance and cardiac computed tomography, also provide important information on LA dynamics but have disadvantages with respect to cost and availability. The current narrative review underlines basic concepts such as the accurate assessment of LAS and discusses the clinical relevance of LAS by pointing out its significant diagnostic and prognostic role in several cardiovascular conditions. The aim of this article is to discuss the current integration of LAS into clinical practice with a view to further improving patient management and treatment strategies.

## 1. Introduction

The assessment of the left atrial strain (LAS) has emerged as a pivotal component in the evaluation of cardiac function, enabling a detailed functional assessment of the left atrium (LA) dynamics throughout the cardiac cycle. Distinct from traditional LA measurements, which focus on LA dimensions, LAS assessment provides detailed information regarding LA function, including its reservoir, conduit, and booster-pump phases. This feature makes LAS a vital parameter for assessing not only structural but also functional cardiac function, particularly in the context of heart failure, atrial fibrillation, valvular heart disease, and myocardial infarction [1]. While speckle-tracking echocardiography (STE) has been the most popular method for analyzing LAS, other advanced imaging techniques such as cardiac magnetic resonance (CMR) and cardiac computed tomography angiography (CCTA) also offer valuable insights [2,3]. Despite its advantages, STE presents several challenges related to image quality, operator skill, and the complexity of LA geometry. These challenges can impact the accuracy and precision of LAS measurements, raising questions about their application in routine clinical practice [3].

In this article, we review the current methodologies for evaluating LAS, inspect the physiological basis and clinical relevance of these measurements, and discuss the limitations and challenges encountered in both research and clinical practice. A comprehensive overview of the current state of LAS assessment is provided as well as its implications for cardiovascular disease management.

## 2. Assessment of LA Strain

**Figure 1:**
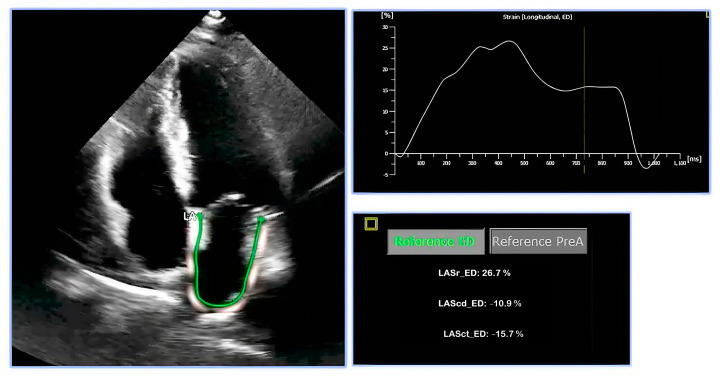
Left atrial strain. The figure shows the different phases of LAS. In the apical four-chamber, begin tracing at the endocardial border of the mitral annulus, follow the LA endocardial border, extending across the pulmonary veins, and/or the orifices of the LA appendage, up to the opposite mitral annulus side. Note, the left atrium is in the far field and, therefore, has reduced image resolution, making it hard to detect speckles. Therefore, we primarily perform contour detection between the blood (black) and the wall (white), followed by calculating length changes. ROI width of 3 mm is recommended, though adjustments should be made to avoid including the pericardium.

**Figure 2:**
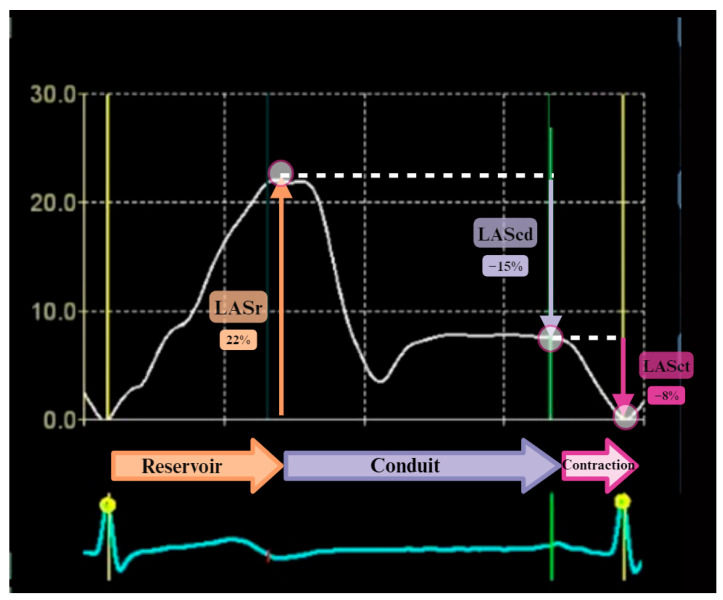
Zero baseline, here defined as the end of diastole. Reservoir—end-diastole to peak (positive valve—LA getting bigger), so LV reservoir function represents LA relaxation and compliance, modulated by LV systolic function through the descent of the LV base. Conduit = value from the peak to the pre-contraction phase (negative value); LA conduit function is reliant on LV diastolic function, including both suction force dependent on LV relaxation and LV chamber stiffness. Contraction = pre-contraction point to baseline (negative value) LA booster function is based on intrinsic LA contractility and LV end-diastolic compliance and pressure.

Left atrial strain assessment provides a detailed picture of LA function throughout the cardiac cycle. It analyzes the three phases of LA activity: reservoir (LA filling by the pulmonary veins during ventricular systole), conduit (passive ventricular filling during early diastole), and booster-pump (active ventricular filling during late diastole) (Figure 1 and Figure 2).

While LA volume assessment is widely recommended by scientific guidelines and represents a traditional marker for LA structural impairment, recognizing the dynamic nature of LA function through LAS assessment is increasingly seen as a more sensitive indicator of cardiovascular diseases [4,5].

Although STE is often the initial modality for analyzing LAS and function, other advanced imaging techniques, such as CMR and CCTA, can also be useful in volumetric and functional analysis of the LA, although the measurements obtained from these different methods may differ. Benjamin et al., in a retrospective study that included 369 patients, demonstrated that LAS measured by CMR was significantly lower in comparison to that measured by STE (15.1 ± 7.1% vs. 18.9 ± 8.3%, mean bias −3.7%, *p* < 0.001). In another retrospective study of 28 participants, a comparison between CCTA and STE revealed a median LAS of 19 (13.5–27.3) using CCTA, compared to 28 (17.5–32.6) when using STE (*p*-value < 0.001) [6,7,8].

### 2.1. Speckle-Tracking Echocardiography

Due to its accessibility and reliability, STE is often the first choice for evaluating LA function and morphology. However, analyzing LA deformation with STE is more complex than analyzing the left ventricle due to the LA’s thin wall, mobile septum, and complex geometry. Moreover, when using a transthoracic approach, the LA’s position in the far field can make achieving optimal signal quality and resolution more challenging.

LAS evaluation using echocardiography requires optimal scan acquisitions for accurate assessment; the imaging sector should be narrowed down to improve the frame rate and resolution while making sure that LA images are not foreshortened. A clear ECG trace with a visible P-wave is crucial, and additional Doppler spectra from the mitral and aortic valves can help in determining time intervals more precisely. The area of interest (ROI) is outlined by programs that provide automatic LA wall detection to make the results reproducible. The ROI usually comprises the endocardial contour of the LA while avoiding the strong pericardial signals, with an optimal width of approximately 3 mm. A careful review of the placement of the ROI is needed; if there are tracking problems, the image may not be suitable for LAS analysis. Strain measurement concentrates on the global longitudinal strain (GLS) of the LA wall with the zero reference normally designated at the end-diastole.

Using 3-dimensional echocardiography (3DE) can address these limitations in assessing LAS, especially LASr, where the traditional approach may overestimate the actual strain due to the foreshortening of LA cavity apical views. Three-dimensional echocardiography datasets provide a comprehensive view of the entire LA, resulting in a more thorough evaluation [5]. Additionally, normal LAS values are lower when measured by 3DE compared to traditional STE [6]. Another potential solution for these limitations is integrating LAS using machine learning algorithms. A study by Carluccio et al. involving 864 patients with heart failure in sinus rhythm demonstrated that the use of machine learning algorithms during LAS assessment can enhance risk stratification in diastolic dysfunction classification over the current recommended guidelines [7]. Future studies evaluating fully automated machine learning techniques to estimate LAS could also be useful in overcoming the current limitations in LAS assessment.

The choice of reference point—P-wave versus R-wave—also plays a significant role in LA function assessment using STE. The P-wave method aligns with LA physiology, accurately showing contraction with negative strain values and more effectively reflecting all three components of LA function: reservoir, conduit, and pump. This method provides reliable data that can distinguish normal from pathologic conditions. In contrast, the R-wave method often overestimates the reservoir and conduit functions and ignores the critical negative peak associated with LA contraction, which can lead to probable misinterpretation in clinical practice. Variations in strain and between these methods complicate the establishment of consistent reference values. Therefore, the P-wave would be the advisable method for its alignment with physiological principles and clinical relevance [8]. However, the R-wave method would be useful in the context of atrial fibrillation.

A standardized protocol for LAS assessment is crucial for ensuring ROI definition, optimal imaging techniques, and accurate interpretation [8]. The strain curve is characterized by a systolic peak and two distinct diastolic phases, with strain calculations of reservoir strain (LASr), conduit strain (LAScd), and contraction strain (LASc) being phasic [9].

LASr represents the difference between the peak strain and the end-diastole, and this effect is positive, whereas LAScd is negative. LASc, which occurs in sinus rhythm, is also negative and represents the difference between the end-diastole and the start of atrial contraction. LA strain measurements integrate left ventricular function and chamber size, providing additional diagnostic and prognostic information. However, the validity and reproducibility of LAS reference values are still under investigation [10].

Automated software has been designed for LAS analysis. Peak atrial longitudinal strain (PALS) demonstrated high reproducibility with both semi-automated and new automated software. This automated software offered better intra- and inter-operator reproducibility, especially in clinic settings with multiple operators, as well as faster analysis. As a result, utilizing automated software to improve the integration of LAS measurements could facilitate its assessment as a part of routine clinical practice, despite ongoing challenges related to standardization and parameter selection [11].

### 2.2. Cardiovascular Magnetic Resonance (CMR)

Currently, cardiovascular magnetic resonance myocardial feature tracking (CMR-FT) is considered the gold standard technique for assessing the morphology and function of cardiac chambers [12]. A wider field of view, low intra- and inter-observer variability, and high tracking quality are among the advantages of CMR-FT over STE [13]. Several limitations, however, can hinder its clinical implementation, including long post-processing times, high costs, limited availability, and challenges associated with patient claustrophobia and metal implants [3]. Nevertheless, despite these constraints, new approaches are still being sought to further optimize and establish the clinical value of CMR. For instance, Leng et al. presented a new semi-automated approach for quantifying long-axis LAS [13]; this significantly reduces processing time and eliminates the need for full contour segmentation of the LA. Instead, this approach relies on the identification of three main anatomical landmarks and yields an average processing time of only 85 ± 10 s per subject, while a manual conventional feature tracking strain analysis required an average of 190 ± 12 s. This may indicate that CMR can deliver timely and precise measurements of LAS and, therefore, enhance the clinical viability of the method.

Despite such advances, clinical uses of CMR for atrial strain currently remain confined to research studies. Of the various factors contributing to this translation lag of such studies into everyday clinical practice, perhaps the most critical factor relates to the lack of uniformity in metrics of strain existing across different CMR vendors. Pathan et al. examined 11 healthy volunteers and 43 patients with clinical indications for CMR to compare the inter-vendor variability in LAS parameters assessed on two more commonly used CMR post-processing software platforms: Medis and CVI (Medis, Medical Imaging Systems, version 2.4.12.2. Leiden, the Netherlands; and CVI version 5.6, Calgary, AB, Canada) [14]. For LASr, the mean LASr by CMR was 39.3 ± 13.8% (Medis: 34.7 ± 11.9%; CVI: 43.8 ± 16.7%) compared to transthoracic echocardiography (TTE), with a mean LASr at 35.0 ± 10.5% (TomTec: 38.4 ± 11.7%; STE: 31.6 ± 9.7%). The inter-vendor intraclass correlation coefficient (ICC) for TTE was 0.86, while for CMR, it was 0.80, with significant mean differences observed (CMR MD 9.1%; TTE MD −6.9%). Regarding LASc, the mean LASc for CMR was 16.1 ± 4.8% (Medis: 14.4 ± 5.0%; CVI: 17.7 ± 5.3%) versus TTE’s mean LASc at 15.3 ± 4.5% (FT: 16.5 ± 5.2%; STE: 14.1 ± 4.3%), with TTE’s ICC at 0.77 and CMR’s at 0.68. For LAScd, the mean LAScd by CMR was 23.2 ± 10.6% (Medis: 20.3 ± 8.8%; CVI: 26.1 ± 13.3%) compared to TTE’s mean LAScd at 19.7 ± 8.1% (FT: 21.9 ± 8.9%; STE: 17.5 ± 7.6%), with TTE’s ICC at 0.89 and CMR’s at 0.79. Reproducibility analysis revealed intra-observer coefficients of variation (COV) for LASr ranging from 6.5% to 12.4% (ICC 0.83–0.95), for LASc from 11.9% to 14.6% (ICC 0.79–0.94), and for LAScd from 8.7% to 13.5% (ICC 0.82–0.92). Inter-observer COVs were 9.9%–13.2% for LASr, 16.8–18.1% for LASc, and 15.1–18.7% for LAScd. These findings highlight systematic differences between CMR and TTE measurements, underscoring the need for calibration and standardized protocols across different modalities and vendors.

These differences were less evident when CMR-FT and volume-gating methods were compared, thus showing that both techniques yield clinically informative measures of LA function. A study by Truong et al. on 123 healthy volunteers reported an LASr of 38.48 ± 9.31%, which was comparable to volume-gating methods (36.77 ± 6.46%; *p* = 0.13), but significantly higher than R-R (32.32 ± 4.89%) and P-P gating (31.60 ± 4.70%; *p* < 0.001) [12]. For LAScd, CMR-FT yielded 24.84 ± 8.29%, significantly exceeding R-R (21.51 ± 5.15%) and P-P (19.16 ± 4.99%; *p* < 0.001) and volume-gating methods (22.05 ± 5.84, *p* = 0.003). LASc was also higher in CMR-FT (13.64 ± 3.96%) compared to R-R (10.82 ± 3.48%), P-P (−12.43 ± 2.81%; *p* < 0.001) and volume-gating methods (14.72 ± 3.73, *p* = 0.03).

Moreover, Truong et al. showed that, while only 81% of the subjects with STE had accurate tracking, CMR-FT achieved excellent tracking in the whole cohort with robust intra- and inter-observer agreement. CMR-FT uses steady-state free precession cine images for CMR to track myocardial strain and thus presents a more comprehensive and reliable way of quantifying LA function. The acquisition protocol usually includes various imaging sequences, such as, but not limited to, short-axis slices and two- and four-chamber long-axis views, to comprehensively capture the entire cardiac cycle.

Although some challenges remain regarding the wider clinical application of CMR for assessing LAS, ongoing improvements in post-processing techniques and an increase in knowledge regarding inter-vendor variability will continue to open new avenues. In that respect, the potential of CMR-FT to provide accurate, reproducible measurements positions this modality well to become a valuable method in the assessment of LA function.

### 2.3. Feature Tracking Computed Tomography (FT CT)

In a study by Hirasawa involving 318 patients aimed at comparing PALS measured by FT CT and STE, a strong inter-modality correlation was observed (correlation coefficient = 0.789, *p* < 0.001). However, PALS_CT_ was significantly lower than PALS_STE_ (15.1 ± 7.1% vs. 18.9 ± 8.3%, mean bias −3.7%, *p* < 0.001). Both modalities demonstrated higher strain values in patients with sinus rhythm compared to those with atrial fibrillation (PALS_CT_: 16.9 ± 6.4% vs. 7.1 ± 3.6%; PALS_STE_: 21.1 ± 7.4% vs. 9.2 ± 4.0%, both *p* < 0.001). The mean absolute percentage error was smaller in sinus rhythm (27 ± 16%) compared to AFib (34 ± 15%, *p* = 0.004). The underestimation of PALS by FT CT compared to STE is attributed to technical differences, including lower temporal resolution (11.8 ± 2.3 frames per second for MDCT vs. 62.0 ± 13.0 frames per second for STE).

Another study on 65 patients by Hosokawa et al. reported that intra-observer ICCs for LASr, LAScd, and LASc were 0.98 (95% CI 0.95–0.99), 0.97 (95% CI 0.95–0.99), and 0.95 (95% CI 0.90–0.98), respectively [15]. Inter-observer ICCs were slightly lower, at 0.94 (95% CI 0.88–0.97) for LASr, 0.90 (95% CI 0.80–0.95) for LAScd, and 0.89 (95% CI 0.77–0.95) for LASc. The previous study, along with other studies [16,17], showed suitable reproducibility for FT CT to measure LAS. This approach can be particularly beneficial when STE evaluation faces challenges due to poor image quality in certain cases. However, the low precision of CCTA raises concerns about reliability [18].

## 3. Physiological Basis of LA Strain

Modulating left ventricle filling is the well-recognized role of the LA, whereas its function as a crucial biomarker of cardiovascular disease is underestimated. Although traditionally it was thought that the best marker for LA dysfunction was its increased size, recent studies have demonstrated that LA function may precede any changes in LA volume and LV function [5,19]. Evaluating LA function can be effectively done by analyzing LAS throughout a cardiac cycle [19].

It is important to note that LAS varies between different sexes and ages. In a cross-sectional study by Stefani et al. involving 147 healthy volunteers, it was observed that LASr and LAScd declined with age. Specifically, LASr decreased from 35.2 ± 6.9 in the general population to 33.8 ± 7.6 in people in their 50s and 32.2 ± 6.0 in people in their 60s (*p*-value = 0.001). Similarly, LAScd decreased from 19.9 ± 6.7 in the general population to 17.7 ± 6.2 in the 50s group and 15.2 ± 4.8 in the 60s group (*p*-value < 0.001). This decline was particularly pronounced among females. LASc would be higher in older populations as a compensatory mechanism for the decreases observed in LASr and LAScd. Additionally, males generally have higher LASc due to this compensatory mechanism [20]. Another study by Boyd et al. showed a notable decrease in LASr and conduit strain from the 6th decade of life, accompanied by an increase in LASc as a corresponding compensatory mechanism [21].

In contrast to these two studies, Meel et al. demonstrated that while LASc remained unchanged in the older population, LA systolic strain showed a declining trend in this age group [22].

Yoshida et al.’s study on sex differences in stroke risk and LA function in patients with atrial fibrillation (Afib) revealed that one of the associations for the higher prevalence of embolic stroke in women might be related to lower LA systolic strain values in females with paroxysmal or persistent Afib [23].

## 4. Clinical Relevance of LA Strain

### 4.1. Role in Heart Failure Diagnosis and Management

Heart failure with preserved ejection fraction (HFpEF) is the most prevalent type of heart failure worldwide, representing over 70% of heart failure cases in older adults [24]. Left atrial remodeling and dysfunction frequently occur in individuals with HFpEF [25,26]. Recent studies suggest that LA dysfunction may play a significant role in the underlying mechanisms of HFpEF, rather than merely serving as a disease indicator. In patients suffering from HFpEF, LA dysfunction is linked to severe symptoms, a higher burden of Afib, reduced exercise capacity, and an elevated risk of morbidity and mortality [26,27,28].

Numerous invasive studies have established a significant correlation between LAS strain and LV filling pressures [29,30,31], as well as a robust association with diastolic dysfunction (DD) [9,32,33,34]. However, the comparative efficacy of LAS versus traditional LA metrics, such as the left atrial volume index (LAVI), in detecting LA abnormalities in heart failure remains ambiguous. In a cross-sectional study involving 473 women from Berlin, an echocardiographic assessment revealed that 29.7% exhibited early-stage diastolic dysfunction (DD1), while 5.0% had advanced-stage DD (DD2). Women with DD1, when compared with women with normal diastolic function (DD0), had reduced LASr and LAScd (DD0, 43.2 ± 8.5% and 27.2 ± 8.0%; DD1, 33.3 ± 8.0% and 16.1 ± 7.1%; *p* < 0.001). However, women with DD1 exhibited higher LA pump function compared to those with normal diastolic function (DD0, 17.6 ± 5.4%; DD1, 18.9 ± 5.5%; *p* < 0.05). On the other hand, all LA functional phases were considerably impaired in DD2 patients, compared with DD0 individuals (reservoir, conduit, and pump function, 29.0 ± 6.3%, 15.1 ± 5.4% [*p* < 0.001], and 14.9 ± 4.1% [*p* < 0.05], respectively) [32]. In another study of 517 patients at risk for left ventricular diastolic dysfunction (LVDD) using 2D speckle-tracking echocardiography, abnormal LAS was found in 62.4% of those with diastolic alterations and high filling pressures, compared to 33.6% with abnormal LAVIs (*p* < 0.01). Adding LAS increased LVDD detection rates from 13.5% to 23.4% (relative increase 73.3%, *p* < 0.01). Abnormal LAS (<23%) was associated with worse functional classes and a high risk of heart failure hospitalization (odds ratio: 6.6). Many patients with normal LAVIs still demonstrated abnormal LAS, indicating that LAS serves as a reliable marker for severe DD (Stage III) across both preserved and reduced LV ejection fractions (LVEFs) [9].

In a comprehensive study by Kagami et al. involving 450 participants, the researchers revealed that during exercise, both LA reservoir and booster-pump strain, as well as compliance, were markedly diminished in patients with HFpEF compared to healthy controls. This impairment in LA function was closely linked to notable limitations in biventricular systolic reserve and the ability to augment cardiac output during physical activity. Furthermore, the study highlighted that these dysfunctions were compounded by abnormal right ventricular–pulmonary artery (RV-PA) uncoupling, which contributed to reduced exercise capacity and an increased risk of worsening heart failure events [35].

The SOCRATES-REDUCED and SOCRATES-PRESERVED trials highlighted the diagnostic value of LAS in HFpEF and heart failure with reduced ejection fraction (HFrEF) [36]. Based on an analysis of a total of 300 patients, 172 (57%) with LVEF < 50% and 128 (43%) with HFpEF, LASr showed greater sensitivity than the LAVI for identifying LA impairment. LASr inversely correlated with the severity of LVDD, indicating a strong link between atrial and ventricular function. LASr also significantly outperformed other metrics like the LAVI, Left Atrial Ejection Force (LAEF), and the mitral E/e′ ratio in predicting severe LV diastolic dysfunction (Table 1 and Table 2)

Notably, its diagnostic value remained independent of LVEF. These findings suggest that LAS should be integrated into clinical assessments for better management of heart failure patients. Furthermore, the previous study found that a more significant reduction in LAS was linked to a decrease in left ventricular GLS (LV-GLS) in patients with HFrEF. However, this association was not observed in those with HFpEF. This suggests that LVDD may function independently of LV-GLS in HFpEF patients.

While much attention has been given to LAS in HFpEF, there are limited studies exploring its prognostic significance in HFrEF [37,38]. For instance, Carluccio et al. found a notable association between declining LAS and poorer clinical outcomes, indicating that lower LAS values were linked to disease progression [37]. Additionally, a study involving 286 HFrEF patients identified LAS as an independent prognostic factor, highlighting the importance of LVDD—beyond just systolic dysfunction—as a marker of worsening cardiac status in those with HFrEF [38].

### 4.2. LA Strain in Atrial Fibrillation

In a single-center retrospective echocardiography study of 126 individuals, including 40 (32%) who developed new-onset Afib (NOAF) and 86 (68%) without NOAF, the authors investigated the relationship between LAS and right atrial strain (RAS), specifically the reservoir phases (LASr and RASr), and the occurrence of NOAF in patients with septic shock. The LASr ROC curve achieved the highest AUC of 0.76 for identifying NOAF at a threshold of 20%, while RASr had an AUC of 0.75 at a threshold of 30%. There was no significant difference between the AUCs (*p* = 0.09). Measurement feasibility was 94% for LAS and 81% for RAS [39]. NOAF is a prevalent complication in this population, associated with increased thromboembolic risk and mortality [40]. The study found that bi-atrial dysfunction, as assessed through STE analysis, significantly correlates with the likelihood of developing NOAF during the early phase of septic shock. These findings suggest that early identification of LA and RA dysfunction may facilitate targeted interventions to mitigate NOAF risk and improve patient outcomes.

In a study involving 956 patients, Olsen et al. investigated the relationship between echocardiographic measures of LAS and subclinical atrial fibrillation (SCAF) by long-term continuous monitoring [41]. The key findings indicated that both reservoir and contraction strain metrics were significantly correlated with SCAF occurrences, even among individuals with structurally normal LA and normal diastolic function. Participants with high LASr (>33%) had an incidence rate of 9.8 events per 100 person-years (95% CI, 8.2–11.8) for SCAF ≥ 6 min, compared to 14.5 for low reservoir function (95% CI, 12.4–16.9). For LASc (>19%), the rates for SCAF were 8.3 (95% CI, 6.9–10.1) for high strain versus 16.2 (95% CI, 14.0–18.8) for low strain.

In a prospective longitudinal study of 4466 participants from the Copenhagen City Heart Study, PALS and peak atrial contraction strain (PACS) were identified as independent predictors of AF during a median follow-up of 5.3 years [42]. Overall, 154 participants (4.3%) developed Afib. PALS had a hazard ratio of 1.05 (95% CI 1.03–1.07, *p* < 0.001) and PACS had a hazard ratio of 1.08 (95% CI 1.05–1.12, *p* < 0.001). Another cohort study of 824 participants assessed LAS and LASr. Over a mean follow-up of 10.9 years, 105 participants (12.7%) developed NOAF. Lower baseline LAS and LASr correlated with new-onset Afib (*p* < 0.01). Adjusted hazard ratios indicated that LAS (HR = 2.05) and LASr during LA contraction (HR 2.24) were significant predictors of Afib [43].

While prior studies have established the clinical significance of LAS in predicting Afib, only a limited number of studies have specifically examined its utility in forecasting SCAF. For instance, a retrospective analysis of 127 patients with pacemakers or implantable cardioverter defibrillators found that a reservoir strain measurement below 37% was associated with a markedly elevated risk of cardiac implantable electronic devices-detected Afib [44]. Similarly, the SURPRISE study found that a reservoir strain of less than 28% significantly increased the risk of SCAF by 5-fold in patients with cryptogenic stroke [45].

Importantly, previous investigations primarily focused on reservoir strain, leaving other echocardiographic strain measures underexplored in relation to SCAF. However, findings from cardiac magnetic resonance imaging in a subset of the LOOP study linked both volume and deformation parameters of reservoir and contractile functions for identifying the occurrence of SCAF [46]. This underscores the potential of LA strain metrics as valuable tools for the early identification of patients at risk for SCAF.

The LA stiffness index is used to assess LA myocardial compliance. Prior studies have shown that this index is higher in patients with persistent Afib and would increase in the setting of Afib radiofrequency ablation but not after pulsed-filled ablation. LAS can be useful in detecting LA stiffness after ablation [47,48,49].

### 4.3. Predictive Value for Cardiovascular Outcomes

A cohort study by Li et al. on 80 patients with end-stage renal disease and HFpEF postulated LA peak circumferential strain of reservoir function (LASr_c) as a prognostic predictor of all-cause mortality or major adverse cardiovascular events (MACEs) in this population. They found that an LASr_c value below 28.5% was associated with higher mortality rates. While they observed an association between LAS reservoir strain, LASc, LAVI, and LAEF with mortality, only LASr_c was an independent predictive factor. It is notable that LA echocardiographic parameters were measured by four-dimensional STE [50].

### 4.4. Association with Myocardial Infarction

Left atrial maximal volume serves as a significant prognostic marker for mortality and heart failure hospitalization post-acute myocardial infarction (AMI). Normal LA volumes indicate favorable outcomes, even with reduced LV function, while enlarged volumes correlate with chronic elevated LV filling pressures and poorer prognoses. Moreover, studies have investigated the role of LAS, in addition to LA volume, in this regard.

In a cohort study involving 1110 patients with AMI, Ersbøll et al. evaluated the prognostic significance of LAS, with a focus on the PALS and its relationship with LV function [51]. The study found that a reduced PALS is closely associated with an increased burden of comorbidities and deteriorating indices of LV systolic and diastolic function. Notably, while PALS demonstrated a relationship with GLS and LA dilatation, it did not provide additional prognostic information beyond established LV function metrics. The findings suggest that LASr, as measured by PALS, is primarily influenced by GLS and LA volume rather than being an independent predictor of adverse outcomes. This challenges the notion that assessing LA deformation offers unique insights in the context of myocardial infarction. The study also highlighted that right ventricular function did not significantly impact LA reservoir function, although diminished tricuspid annular plane systolic excursion was observed alongside reduced PALS, likely confounded by impaired GLS due to extensive LV myocardial damage.

On the other hand, a study by Zhang et al. on patients with ST-Elevation Myocardial Infarction (STEMI) who underwent primary percutaneous coronary intervention (PCI) and completed CMR imaging within a week revealed different results [52]. This study revealed that LA longitudinal strain is a critical prognostic indicator post-STEMI, with LAScd serving as an independent predictor of MACE. Notably, the combination of LAScd and LV-GLS enhanced MACE prediction, highlighting that those patients with concurrent impairments in both strain values faced the most adverse outcomes. Further studies are needed to shed light on this matter.

### 4.5. LA Strain in Diabetes Mellitus

Diabetes mellitus, with a prevalence of 9.3% of the global population (463 million individuals) as of 2019 [53], is associated with structural changes in the heart and impaired LA function [54]. An investigation involving 134 participants, with 66 diagnosed with type 2 DM (T2DM) and 68 healthy controls, revealed statistically significant reductions in LASr (31.2% ± 4.56%), LAScd (14.77% ± 6.3%), and contractile functions (16.36% ± 4.82%) among T2DM patients compared to controls (LASr: 38.75% ± 5.43%, LAScd: 19.58% ± 5.91%, and contractile function: 19.16% ± 4.98%, respectively) [55].

Another study evaluated LA function using STE in 155 patients with hypertension and/or diabetes. It was observed that PALS was significantly lower in diabetic patients (24.7 ± 6.4%) and those with hypertension (29.0 ± 6.5%), and the lowest levels were found in patients with both conditions (18.3 ± 5.0%) compared to controls (39.6 ± 7.8%). This suggests that the combined impact of these cardiovascular risk factors worsens LA dysfunction [56].

In patients with HFpEF, particularly those with T2DM, a higher prevalence, severity, and duration of diabetes were notably correlated with lower PALS values [57]. This indicates that PALS may serve as a valuable prognostic marker for identifying HFpEF patients at increased risk for adverse outcomes related to T2DM, warranting more aggressive therapeutic strategies.

### 4.6. LA Strain and Stroke

Previous studies have demonstrated that LAS may improve stroke risk stratification in older adults. A systematic review and meta-analysis that included six studies involving 3587 patients with Afib found that 361 patients (10.1%) experienced a cerebrovascular accident (CVA) [58]. Decreased LASr was identified as a predictor of CVA in patients with AF, with an odds ratio of 0.88 (95% CI: 0.81–0.96, *p* = 0.005).

In addition, a cohort study involving 806 adults aged 55 years or older with a mean follow-up of 10.9 years found that individuals with ischemic stroke exhibited lower baseline positive longitudinal LAS and negative longitudinal LASr compared to those without stroke [59]. Multivariable analysis showed that the lowest quintile of positive LAS had an adjusted HR of 3.12 (95% CI, 1.56–6.24), while the lowest LASr had an HR of 2.89 (95% CI, 1.44–5.80) linked to stroke incident risk. In participants with normal LA size, the HR for positive LAS was 4.64 (95% CI, 1.55–13.89), and for negative LASr it was 11.02 (95% CI, 3.51–34.62).

Another study suggests LASr as a useful marker for distinguishing between cardioembolic and non-cardioembolic etiology of CVA. The study analyzed 418 consecutive patients with ischemic stroke or transient ischemic attack, comprising 229 with cardioembolic and 189 with non-cardioembolic stroke etiology. The study found that LASr was significantly more impaired in those with cardioembolic strokes (16.7 ± 8.2%) compared to non-cardioembolic strokes (26.0 ± 5.5%), with a *p*-value of <0.01. LASr showed excellent discrimination in distinguishing stroke subtypes (AUC 0.813, 95% CI 0.773–0.858), outperforming LVEF, LAVI, and E/e′ with significant differences (AUC differences ranging from 0.083 to 0.163, all with *p* < 0.01) [60].

### 4.7. LA Strain in Kidney Diseases

As cardiovascular disease is the most common cause of death in patients with reduced eGFR, the identification of risk factors for cardiovascular deaths in these populations has become essential [61]. In a study involving 243 stage 3/4 CKD patients without prior cardiac disease, participants were monitored for 3.9 ± 2.7 years. The primary outcome was cardiovascular death or MACE, which occurred in 54 patients. Key independent predictors for cardiovascular death and MACE included older age, diabetes mellitus, anemia, greater LV mass, reduced LV-GLS, larger indexed LA volume, higher E/e′ ratio, and reduced LASr, with *p* < 0.01 for all. LASr (*p* < 0.01) emerged as the sole independent predictor for both endpoints in multivariable analysis. ROC analysis indicated LASr had a stronger predictive ability (AUC = 0.84) compared to the Framingham (AUC = 0.58) and Atherosclerotic Cardiovascular Disease (AUC = 0.59) risk scores [62].

A cross-sectional study involving 169 patients with chronic kidney disease (CKD) in Vietnam from April to November 2022 assessed patients with CKD stages 1 through 5. Increased CKD severity correlated positively with LV diastolic/systolic diameters, LV mass, E/e′ ratio, and maximal tricuspid regurgitation velocity; however, it was negatively correlated with LV-GLS. Significant associations emerged between LV mass (β = 0.068), ejection fraction (β = 0.112), LASr (β = −0.077), and eGFR reduction. However, LAScd and LASc were not found to be significantly correlated with eGFR reduction (*p* > 0.05) [63].

As mentioned in Section 4.3, LASr_c is a significant prognostic predictor of all-cause mortality and MACEs in patients with end-stage renal disease and HFpEF [50].

### 4.8. LA Strain and Valvular Heart Diseases

There are relatively fewer studies that have evaluated LAS and valvular heart diseases. One of these was a study of 220 patients with bicuspid aortic valve and significant aortic regurgitation. The primary endpoint was a composite of all-cause mortality, heart failure hospitalization, and aortic valve repair or replacement. A LASr threshold of <24% was considered as LA dysfunction. During a median follow-up of 364 days (interquartile range of 294 to 752 days), 46 patients (20.9%) reached the composite endpoints. Multivariable Cox analysis revealed that impaired LASr (adjusted hazard ratio, 2.08 [95% CI, 1.05–4.11]; *p* = 0.036) was a significant predictor of these endpoints after adjustment for other predictors [64].

In a study of 159 patients with severe Carpentier II mitral regurgitation (MR) (LVEF ≥ 60%), 55 had follow-up echocardiography with a mean follow-up of 15.3 ± 8.3 months [65]. A poor baseline LASr rate was associated with older age, LA elongation, increased LAVI, and reduced left ventricular strain (*p* < 0.001). Only the change in LASr rate (OR 0.037; CI 0.003–0.496, *p* = 0.013) predicted accelerated LA remodeling, correlating with significant deterioration of LA deformation (β = −0.424, *p* = 0.002). Another study evaluated the application of LAS as a guide to the timing of intervention in mitral valve prolapse (MVP) in 192 patients [66]. The Time-Lapse Atrial Ejection Fraction (TLAEF) was identified as a significant predictor (OR 0.78; 95%CI: 0.68–0.89, *p* < 0.001). LASr also proved to be a significant predictor, with an OR of 0.91 (95% CI: 0.83–0.99, *p* = 0.028), as did LASc (OR 0.86, 95% CI: 0.75–0.98, *p* = 0.021). However, LAScd was marked as a non-independent predictor (OR 0.92; 95% CI: 0.82–1.03, *p* = 0.128). The ROC curve performance highlighted TLAEF, with an AUC of 0.96, LASr at 0.90, and LASc at 0.89, indicating excellent predictive ability for the need for mitral valve surgery.

Another study was conducted by Figueiredo et al. [67] involving 493 patients with mitral stenosis (MS) (mean valve area 1.1 ± 0.4 cm^2^, 84% female). Impaired LAS was evident in all patients at baseline, with an LASr of 12.5 ± 7.1%, LAScd of 6.7 ± 4.1%, and LASc of 6.0 ± 4.2%. At baseline, 166 patients (34%) had been diagnosed with Afib, and 62 more patients (19%) developed Afib during the follow-up period. This study demonstrated that LASr can act as an important predictor of new-onset Afib, using a cut-off of 17.9%, as those with LASr ≤ 17.9% had a significantly higher incidence of new-onset AF compared to those with LASr > 17.9% (24.3% versus 4.5%, *p*-value < 0.001). Over a mean follow-up of 3.8 years, 125 patients (25%) required mitral valve replacement, and 32 patients (6.5%) died, with a higher rate of adverse effects observed in those with new-onset Afib (56%).

In a cross-sectional study [68] involving 666 patients (mean age 66 ± 11 years; 68% male), with the majority exhibiting severe MR (82%), the median LASr was 9.8% (IQR, 6.6%–14.5%). The multivariable analysis revealed that more severe MR was independently associated with impaired LASr (odds ratio 0.419; 95% CI, 0.249–0.704; *p* = 0.001). During a median of 5 years of follow-up, 58% of patients died, with significantly higher mortality in patients with LASr < 9.8% in comparison to those with LASr ≥ 9.8% (85% vs. 45% at 5 years; *p* < 0.001). Additionally, LASr maintained its significance as a prognostic indicator for all-cause mortality in patients with MR after multivariable adjustment (HR 0.499; 95% CI, 0.386–0.645; *p* < 0.001).

A cohort study [69] included 87 patients (median age 60 years, 70.1% male) who underwent either a MitraClip placement (*n* = 49) or mitral valve replacement (MVR) (n = 38). After 12 months of follow-up, all MVR arms achieved MR ≤ 2+, while 13 patients in the MitraClip arm had 0 or 1+ MR. At baseline, LAS results were similar in the MitraClip and MVR groups. Moreover, in patients with reduced baseline LAS, no significant change in LAS was observed in either group. Correlations were observed between baseline and follow-up LAS and longitudinal LV strain (LVS) (r = −0.28; *p* = 0.01 and r = −0.37; *p* < 0.001, respectively). Notably, changes in LAS were related to changes in LVS (r = −0.24; *p* = 0.03).

## 5. Current Guidelines and Standards and Technological Advances and Innovations

Due to a lack of standard techniques and guidelines for assessing the LA function and less familiarity with ultrasound methods for imaging the LA, the systemic investigation of LA function is usually not performed. Nowadays, with increasing interest and understanding of the importance of the identification of LA dysfunction, new echocardiographic techniques can help cardiologists to diagnose LA dysfunction, which is anticipated to provide a new perspective on the clinical management of several cardiac diseases.

Strain Doppler, speckle-tracking, and 3D echocardiography are some of these new techniques in assessing the LA function [70]. No single parameter can fully define LA function, but various measures have been proposed, including the LA function index (LAFI) and volumetric measures like LAEF and the LA expansion index [19,71,72,73].

### 5.1. Echocardiographic Methods in Patients Without Sinus Rhythm

#### 5.1.1. Left Atrial Function Index (LAFI)

The LAFI is a comprehensive and rhythm-independent index that combines the effects of cardiac output, the ability of the LA to act as a reservoir, and its size. This index is based on the principle that reduced cardiac output, impaired atrial reservoir function, and increased atrial size are associated with lower atrial function [74].

#### 5.1.2. Left Atrial Ejection Fraction (LAEF)

LAEF is a measure to gauge how effectively the LA pumps blood into the LV at the end of the ventricular diastole and demonstrates the LA systolic function. It is calculated using Newton’s second law, in which the force exerted by the LA is equal to multiplying the mass of the blood by its acceleration [74].

#### 5.1.3. Left Atrial Expansion Index (LAEI)

The LAEI is a valuable parameter used in echocardiography to assess the LA function in patients without sinus rhythm. Two-dimensional STE is utilized to evaluate LAS in these patients. The LAEI can improve diagnostic accuracy, especially in distinguishing between different types of diastolic dysfunction [19,71,72,73].

## 6. Discussion

The assessment of LAS has gained much attention in clinical settings as an important tool for cardiac function assessment, especially but not only regarding heart failure and atrial fibrillation. However, this methodology also encounters some critical gaps, challenges, and limitations that significantly hinder its implementation. Among the most relevant limitations of STE—the most common modality applied for measuring LAS—are the variability in image quality. These could be a result of suboptimal acoustic windows and patient-specific characteristics, leading to inconsistent findings. Additionally, anatomical plane limitations limit STE, often necessitating multiple views in order to obtain accurate measurements. Such complexity may, therefore, lead to a lack of inter-observer agreement, thus challenging the implementation of LAS measurement in clinical and research fields. This, in turn, may further shift reliance toward other modalities, such as CMR and CCTA, for the assessment of left atrial function (Table 3).

In response to these challenges, the integration of AI combined with 3DE is a promising frontier. Studies, such as the recent one by Nabeshima et al. [75] and the fifth Copenhagen City Heart Study [76], are instrumental in advancing this technology toward clinical practice. However, the broader adoption of this technology has been hindered by the absence of standardized protocols and clinician training. Future research, including prospective validation cohorts, should aim to address these limitations.

Another significant challenge in the evaluation of LAS is the complexity involved in interpreting its functional parameters. Although emerging research demonstrates the capability of machine learning models in integrating LAS into protocols and algorithms, no consensus has yet been reached as to the precise role of LAS parameters. Moreover, most of the literature now is focused on LAS in specific conditions, such as in HFpEF and AFib, with limited exploration of its prognostic value in other conditions, such as HFrEF. While a few studies have investigated LAS across different conditions, further research is needed to establish LAS as a clinical marker for these specific diseases. For instance, in myocardial infarction, findings remain inconsistent: Zhang et al. [52] reported that LAScd was independently predictive of MACEs, while Ersbøll et al. [51] suggested that PALS may not offer additional prognostic value beyond established metrics of LV function and LA volume. Such differences again highlight the need for uniform measurement protocols and further prospective studies on larger cohorts to interpret the role of LAS in various cardiac conditions. Moreover, while there is evidence to suggest that LAS may have a role in the stratification and prognosis of stroke risk in diabetes, the overall evidence remains limited. LAS may serve as a critical marker of cardiovascular diseases in diabetes mellitus, especially those with HFpEF, but comprehensive longitudinal studies are imperative to identify underlying causal relationships and determine how LAS might be integrated into the clinical management of diabetes-related cardiovascular risk.

Current research and clinical practice face limitations due to variations in study populations and methodologies. The generalizability of the findings is often hindered by small sample sizes or the use of specific patient cohorts. Furthermore, the absence of a standardized protocol for measuring LAS complicates the comparison of results across different studies and clinical settings.

Patient-specific factors also play a crucial role in the accuracy of strain evaluation. Age, body habitus, and underlying cardiovascular conditions can affect image quality during echocardiography and the precision of the strain measurement. For example, in a patient with obesity and advanced heart failure, image quality can be jeopardized, leading to less reliable LAS measurement. To address these challenges, ongoing advancement in echocardiographic technology and more consistent standardization efforts and guidelines are necessary.

This could enhance the reliability and applicability of LAS measurements in both clinical practice and research settings.

## 7. Conclusions

Evaluating LAS has become increasingly important for understanding cardiac function, offering insights beyond traditional LA volume measurements. This review highlights LAS’s crucial role in the diagnosis and management of many cardiovascular diseases, and the valuable prognostic information offered.

Despite its benefits, current LAS measurement methods face limitations, including image quality issues, technique variability, and patient-specific factors. Enhancement of echocardiographic technology and standardized protocols are required to improve the reliability and clinical outcomes. Future research should focus on refining these techniques and establishing consistent guidelines for LA function assessment.

## Figures and Tables

**Table 1:** Area under curve (AUC) and performance characteristics for cardiac function parameters.

Parameter	AUC	95% CI	Cut-Off	Sensitivity	Specificity
LASr	0.83	0.77–0.88	14.1%	80%	77.8%
LAVI	0.66	0.59–0.73	41.6 mL/m^2^	65.7%	60%
LAEF	0.70	0.59–0.73	40.1%	69.2%	65.7%
E/e′ average	0.60	0.53–0.68	14	53.6%	58.1%

**Table 2:** AUC Comparison by DeLong’s Method.

Comparison	*p*-Value
LASr versus LAVI	<0.01
LASr versus LAEF	<0.01
LASr versus E/e′ average	<0.01

**Table 3:** Comparison of different imaging modalities for assessing LAS.

	STE	CMR	CCTA
**Advantages**	Accessible Reliable	Gold standard	Accessible Can assess coronary arteries
**Disadvantages**	Variable imaging quality Limitation from anatomical planes Subjective	Expensive Not available Has contraindications	Not precise
